# Recent Developments in DNA-Nanotechnology-Powered Biosensors for Zika/Dengue Virus Molecular Diagnostics

**DOI:** 10.3390/nano13020361

**Published:** 2023-01-16

**Authors:** Goeun Park, Hanbin Park, Sang-Chan Park, Moonbong Jang, Jinho Yoon, Jae-Hyuk Ahn, Taek Lee

**Affiliations:** 1 Department of Chemical Engineering, Kwangwoon University, Seoul 01897, Republic of Korea; 2 Department of Electronics Engineering, Chungnam National University, 99 Daehak-ro, Yuseong-gu, Daejeon 34134, Republic of Korea; 3 Department of Biomedical-Chemical Engineering, The Catholic University of Korea, 43 Jibong-ro, Wonmi-gu, Bucheon-si 14662, Gyeonggi-do, Republic of Korea; 4 TL Bioindustry, 20 Kwangwoon-ro, Nowon-gu, Seoul 01897, Republic of Korea

**Keywords:** ZIKV, DENV, DNA nanotechnology, electrochemical biosensor, electrical biosensor, optical biosensor

## Abstract

Zika virus (ZIKV) and dengue virus (DENV) are highly contagious and lethal mosquito-borne viruses. Global warming is steadily increasing the probability of ZIKV and DENV infection, and accurate diagnosis is required to control viral infections worldwide. Recently, research on biosensors for the accurate diagnosis of ZIKV and DENV has been actively conducted. Moreover, biosensor research using DNA nanotechnology is also increasing, and has many advantages compared to the existing diagnostic methods, such as polymerase chain reaction (PCR) and enzyme-linked immunosorbent assay (ELISA). As a bioreceptor, DNA can easily introduce a functional group at the 5′ or 3′ end, and can also be used as a folded structure, such as a DNA aptamer and DNAzyme. Instead of using ZIKV and DENV antibodies, a bioreceptor that specifically binds to viral proteins or nucleic acids has been fabricated and introduced using DNA nanotechnology. Technologies for detecting ZIKV and DENV can be broadly divided into electrochemical, electrical, and optical. In this review, advances in DNA-nanotechnology-based ZIKV and DENV detection biosensors are discussed.

## 1. Introduction

The Zika virus (ZIKV) is a mosquito-borne virus in the genus Flavivirus, which also includes the yellow fever virus and West Nile virus. It can be vector-borne by mosquitoes infected with ZIKV and can be transmitted through non-vector transmission, such as sexual contact or blood transfusion [1,2]. Furthermore, it can be transmitted from the mother to the fetus, causing microcephaly [1,2]. ZIKV infection is often misdiagnosed or undetected [1,3], and it has been reported that approximately 80% of infected people are asymptomatic, while others show mild symptoms, such as joint pain and fever [1,4].

ZIKV was first isolated in 1947 from rhesus monkeys in Uganda [5], and the first human case of infection was reported in Nigeria in 1954 [6]. When the first ZIKV infection occurred on Yap Island, approximately 73% of the population were infected and approximately 18% of them were symptomatic [7]. Since then, cases of ZIKV infection have been reported in the Cook Islands [8], New Caledonia [9], French Polynesia [8], Easter Island [10], and more recently, in the United States [11] and Europe [12].

The dengue virus (DENV), like ZIKV, is a mosquito-borne virus belonging to the genus Flavivirus. Mosquitoes infected with DENV infect humans, and four different serotypes (DENV-1, DENV-2, DENV-3, and DENV-4) are antigenically distinct so that antibodies cannot cross-neutralize [13,14]. Approximately 80% of those infected with DENV are asymptomatic, whereas others show symptoms, such as fever, headache, and vomiting, as well as a rash that appears 3–4 days after the onset of fever [13]. However, plasma leakage may occur during secondary infections, leading to circulatory disturbances, shock, and death [15]. It has been reported that DENV surged worldwide between 1960 and 2010 due to various issues, such as global warming, population growth rate, inefficient mosquito control, and lack of medical facilities [16,17].

The first case of a dengue-like disease was reported in Madras in 1780, while the first virologically documented case of DENV was reported in India in 1963 [16]. Approximately 2.5 billion people live in DENV-affected areas, with roughly 400 million infections per year, and mortality rates in some areas have been reported to exceed 5–20% [17,18]. In 2001, 69 countries reported DENV outbreaks, and in 2002, more than 1 million cases were reported in the Americas alone [17]. Recently, the area where DENV generally occurs has expanded, and has now been reported in Asia, Africa, and the Americas [17].

The resumption of globalization after the pandemic and an increase in overseas travelers are predicted to increase the number of mosquito-borne viruses imported from abroad. Due to global warming, the vector ecosystem is changing, and the number of travelers is increasing along with urbanization and changes in crowd immunity [19]. In 2017, Brazil declared the end of the ZIKV epidemic [20]; however, in the same year, it was reported that the epidemic was highly likely to occur in India [19]. In 2014, it was reported that DENV was prevalent in Southeast Asia and Guangzhou, China [21]. As such, there is a high probability that ZIKV and DENV are prevalent worldwide. In addition, simultaneous infection with ZIKV and DENV is very dangerous due to the occurrence of transient anemia and acute tissue damage [22], and DENV antibodies can enhance ZIKV infection [23]. Therefore, it is necessary to establish a method for the accurate diagnosis of ZIKV and DENV infections.

The diagnostic methods for ZIKV and DENV recommended by the World Health Organization (WHO) thus far include reverse transcriptase polymerase chain reaction (RT-PCR), enzyme-linked immunosorbent assay (ELISA), and plaque reduction neutralization tests (PRNT) [24,25]. However, these diagnostic methods are often unsuitable for on-site analysis where rapid diagnosis is required due to expensive equipment and limited resources [26,27]. Since RT-PCR requires the previous step of polymerase chain reaction (PCR) to generate DNA from viral RNA, it takes a long time to confirm the diagnosis [28]. ELISA causes cross-reactivity between antibodies from different ZIKV and DENV viruses, which have similar target protein sequences, making it difficult to diagnose accurately [29,30]. PRNTs are used to discriminate antibodies from closely related viruses [29]. However, PRNT requires reagents that are not commonly available, require a long time to confirm results, and are expensive [29,31]. Therefore, there is a need for a diagnostic method that incorporates DNA nanotechnology to compensate for these shortcomings.

**Figure 1:**
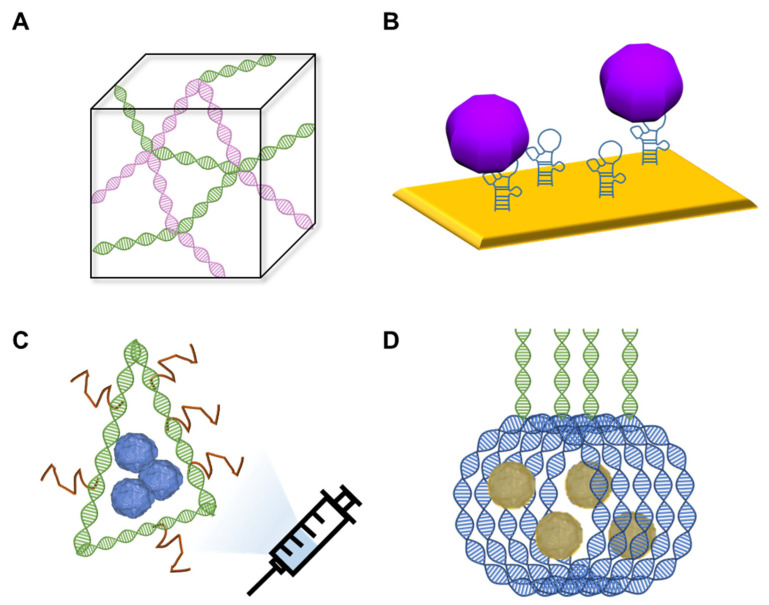
Application of DNA nanotechnology in (**A**) DNA hydrogel, (**B**) biosensors, (**C**) vaccines and (**D**) drug delivery.

Recently, DNA nanotechnology has been applied to various fields. DNA nanotechnology is a technique used for the design and manufacture of artificial nucleic acid structures for technological applications. Because DNA nanostructures have high editability, they can be designed and synthesized with excellent predictability at the nanoscale using DNA nanotechnology [32]. Studies of this technology have been reported for DNA hydrogels [33] biosensors [34,35,36,37,38], vaccines [39], and drug delivery [40] (Figure 1). Hydrogel is a polymer with a three-dimensional structure that contains a large amount of water and is used in various fields such as biomedicine and drug delivery [41]. DNA hydrogel technology integrates the biological function of DNA and the skeletal function of the hydrogel, and as a polymer, DNA enhances the function of the hydrogel [42]. DNA is embedded in a polymer hydrogel that has excellent biodegradability, biocompatibility, and permeability [43]. DNA nanotechnology can amplify the output signal using the DNA-specific chain reaction in the biosensor, thereby meeting the demand for low detection limits [44]. In addition, two-dimensional and three-dimensional DNA nanostructures can encapsulate vaccine antigens and biochemical molecules [39]. Moreover, they have a number of advantages, such as the reduction of drug toxicity and enhancement of drug targeting, and have high potential as powerful and simple design techniques for the self-assembly of nanostructures [45]. Among these, the most noteworthy is the DNA-nanotechnology-based biosensor. DNA-nanotechnology-based biosensors are based on the principle that DNA nanostructures bind to target materials, and unlike biosensors based on antibodies or enzymes, they can be manufactured at low cost and with high assembly efficiency and have high sensitivity [46]. This induces a conformational change in the DNA nanostructure, which is used as a signal readout [44,47]. Sensible stimuli include viruses [37], cancer cells [36], proteins [35], nucleic acids [34] and molecules [31]. Currently, research using single-stranded DNA (ssDNA) as a bioreceptor is being actively conducted. It is developed to specifically bind the nucleic acid of a target material, and a process of comparison with a material having a similar nucleotide sequence is required. Recently, a DNA aptamer used in a DNA-nanotechnology-based biosensor for virus detection has attracted attention as a bioreceptor that may replace antibodies for the detection of a target substance.

DNA aptamers are produced through systematic evolution of ligands by exponential enrichment (SELEX) and specifically bind to target substances [48]. Aptamers can recognize specific molecules through chemical bonds, including H-bonds, pi-pi, van der Waals, and hydrophobic interactions. Recently, biosensors detecting toxic contaminants have been reported. As bioreceptors, molecular structure switching, target-induced displacement, G4-quadruplex-assisted label-free detection, sandwich structures, split aptamers, and nanoparticle-conjugated aptamers have been suggested [49]. As a bioreceptor, antibodies are difficult to produce, costly to produce, sensitive to temperature and have a short lifespan [50]. Conversely, DNA aptamers are made through an in vitro process, which is economical and has high-temperature stability, thus mitigating the shortcomings of antibodies [48]. In addition, novel DNA aptamers can be easily developed due to their unique properties, such as nucleic acids, convenience of structural design, and high flexibility of the structure [48,51]. In addition, DNA three-way-junction (3 WJ) [52] and DNA four-way-junction (4 WJ) [53] can be prepared using DNA aptamers and used as bioreceptors. Furthermore, virus detection biosensors, using various DNA nanotechnologies, such as DNA origami [54] and DNAzyme [55], are being developed. In addition, this technology can be applied to sensor technology that can detect human chorionic gonadotropin (hCG), a protein produced by the mother’s placenta to determine whether she is pregnant and to detect proteins and nucleic acids of viruses such as COVID-19.

Biosensors are portable and easy to handle; therefore, they can be used directly in the field [56]. Methods for detecting target substances using biosensors include electrochemical [57,58], electrical [59,60], and optical methods [61]. Furthermore, various virus detection biosensors based on DNA nanotechnology have been developed. In this review, we discuss DNA-nanotechnology-based ZIKV and DENV detection biosensors using three detection methods.

## 2. Electrochemical-Based Detection

**Figure 2:**
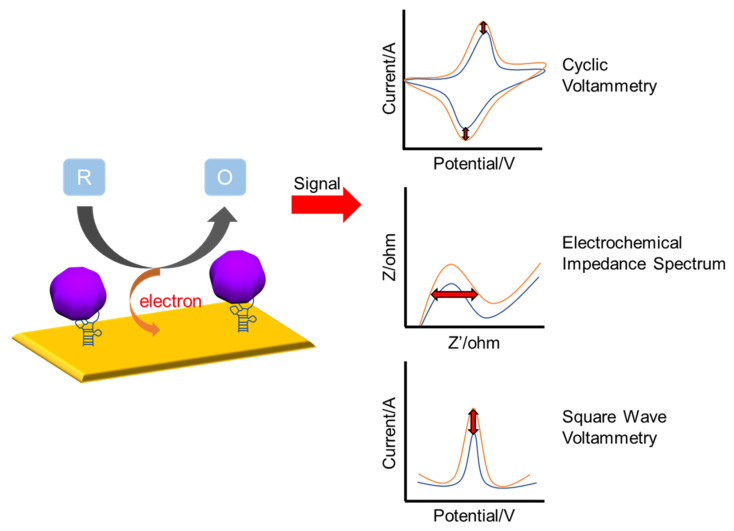
Schematic diagram of an electrochemical sensor. Changes in CV, EIS, and SWV signals before and after binding.

The electrochemical sensor quantitatively detects the current generated through the oxidation and reduction reactions of specific chemical species occurring on the electrode surface [62,63,64]. Electrochemical-based biosensors are being applied in clinical and biological fields, as well as diagnostic medicine and biomedical engineering through the analysis of bio-targeted materials [65,66,67]. It has been confirmed that the electrochemical biosensor has advantages such as high sensitivity, machine miniaturization, fast response time, high selectivity, and low cost. In addition, electrochemical sensors based on DNA and aptamer technology have been suggested for use in various fields, such as immunology and health monitoring [68,69,70,71,72,73,74]. Electrochemical measurement methods can be divided into three main groups depending on the pulse waveform to which voltage is applied [75]; cyclic voltammetry (CV) [76] and square wave voltammetry (SWV) [77]. CV, the method introduced in this section, is a technique commonly used for the characterization of redox reactions on the electrode surface [78,79]. This technique, controlled by an electrochemical workstation, measures the potential between the working electrode and the counter electrode in the cycle phase, increases linearly with time, and measures the current generated by applying a triangular wave voltage [57,80,81,82]. Electrochemical impedance spectroscopy (EIS) is a powerful tool used for probing the electrode surfaces. Impedance indicates the degree to which the electrical flow is interrupted for alternating current, and the electrode surface is analyzed using the characteristics of amplitude change and phase change according to the frequency [83,84,85,86,87]. Figure 2 shows a schematic of the electrochemical measurements. In this section, we discuss DNA-technology-based ZIKV and DENV detection electrochemical sensors.

**Figure 3:**
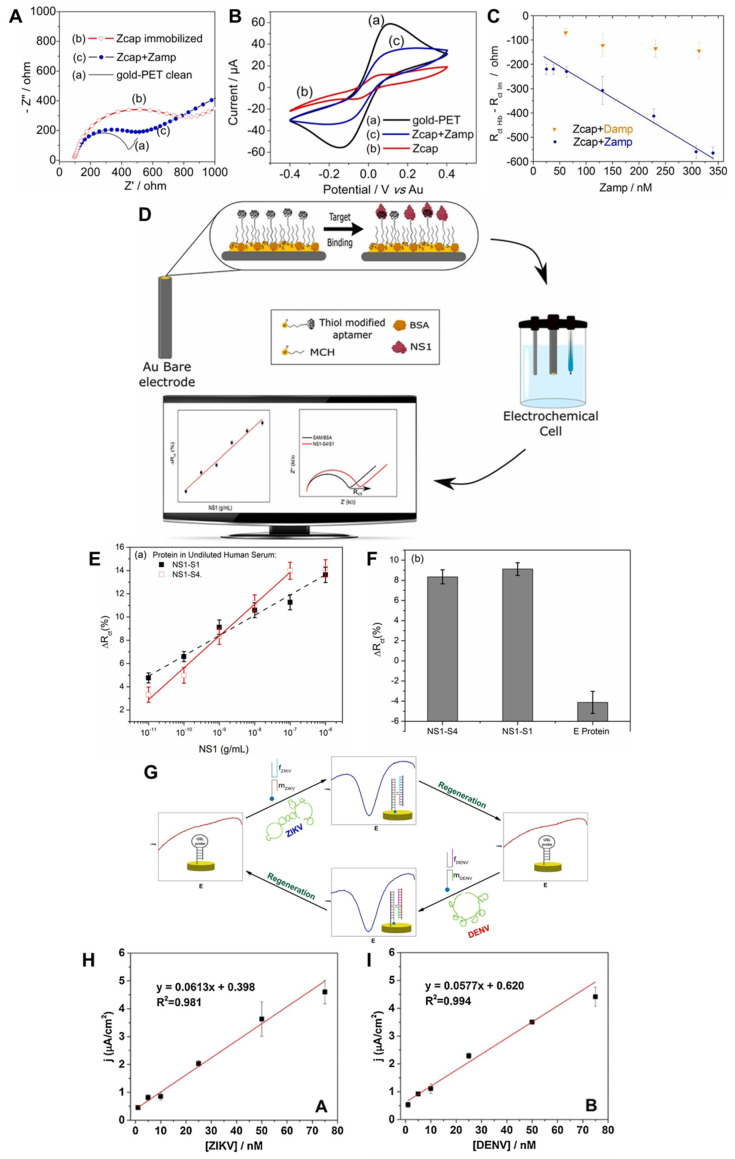
DNA−technology−based ZIKV and DENV detection method using electrochemical method. (**A**) Nyquist diagram showing the three−step impedance behavior of the gold−Polyethylene terephthalate (PET) biosensor: (a) Clean electrode, solid black line; (b) Z_cap_ (capture sequence) immobilization; (c) Z_amp_,(complementary sequence) hybridization full blue circle (Z_cap_ immobilized at 0.40 μM for 6 h at a Z_amp_ concentration of 130 nM). (**B**) Cyclic voltammetry for three steps of gold-PET biosensor: (a) Clean electrode, black curve; (b) Immobilization of the capture sequence (Z_cap_), red curve; (c) Hybridization, blue curve (immobilization of 0.40 µM Z_cap_ for 6 h at 45 °C and a Z_amp_ concentration of 130 nM). (**C**) Analytical curve of charge transfer resistance (ΔR_ct_ = R_ct_ Hib − R_ct_ Im). A total of 0.40 μM fixation of the capture sequence (Z_cap_) for 5 h. Hybridizations with negative controls using Z_amp_ at concentrations of 25, 38, 63, 130, 228, 308 and 340 nM (blue circles) and D_amp_ at concentrations of 63, 130, 228 and 308 nM (orange triangles). Reproduced with permission from [88], published by Elsevier, 2019. (**D**) Schematic diagram of the electrochemical aptasensor for NS1 detection. (**E**) Analytical curves comparing biosensor performance for NS1-S1, S4 in undiluted human serum (standard deviations for at least 3 individual electrodes). (**F**) Changes in charge transfer resistance (ΔR_ct_) of undiluted human serum NS1-S4, NS1-S1 and envelope protein at a concentration of 1 ng/mL for other DENV proteins. Reproduced with permission from [89] published by Elsevier, 2021. (**G**) Schematic diagram of a platform for the detection of two different DNA sequences (ZIKV, DENV) using one electrochemical sensor. (**H**) Calibration curve of ZIKV sensor response (current density) at a 10 min response time using T-ZIKV of the corresponding concentration with m−ZIKV and f−ZIKV. (**I**) Calibration curve of DENV sensor response at a 30 min response time using T−DENV of the corresponding concentration with m−DENV−11 and f−DENV−19. (concentration range 1 nM–75 nM). Reproduced with permission from [90], published by Elsevier, 2019.

In a recent study, Faria et al. developed a label-free polyethylene-terephthalate-based electrochemical DNA biosensor for ZIKV [88]. For the capture probe DNA, forward and reverse primers were selected within the gene sequence encoding the ZIKV NS5 nonstructural protein. The capture probe sequence is identical to the forward primer but functionalized with a thiol group at the 5′ end and complementary to the 3′ end of the target sequence. Through the three-step Nyquist diagram of the biosensor in Figure 3A, it was confirmed that the formation of a double helix together with the capture DNA immobilized on the biosensor is promoted through target DNA denaturation. However, it was confirmed that the impedance was greater than the initial impedance of the pure electrode. Figure 3B shows the three-step CV results of the biosensor. This was confirmed by an increase in the redox peak in the hybridization step of Z_amp_ after the capture sequence of Z_cap_. Figure 3C displays the analytical curve of charge transfer resistance comparing the selectivity of the fabricated sensor to ZIKV and the selectivity of DENV. Damp and Z_amp_ are ΔR_ct_ values hybridized with non-complementary (DENV) and complementary (ZIKV) sequences, respectively. A change in ΔR_ct_ with a concentration change in the concentration range (63, 130, 228, 308 nM) was observed only in the complementary hybridization bond, Z_cap_ + Z_amp_, and the LOD was measured to be 25.0 ± 1.7 nM. Thus, the developed biosensor exhibited selectivity for ZIKV in the synthetic DNA analysis, proving its potential for clinical analysis.

Junior et al. developed an electrochemical biosensor using a DNA aptamer in order to detect nonstructural protein 1 (NS1), which is a major biomarker of DENV [89]. The fabricated aptamer-based sensor provided fast response time, low cost, and high selectivity against dengue fever. Figure 3D is a schematic diagram of the study, where a self-assembled monolayer was completed by immobilization with an aptamer and 6-mercapto-1-hexanol (MCH) on a gold electrode and was made non-specific by adding bovine serum albumin to the NS1 solution. It was stabilized by preventing interaction and the performance of the fabricated biosensor was tested with a human serum solution of NS1 protein serotypes 4 and 1 and measured using electrochemical impedance spectroscopy (EIS). Figure 3E demonstrates a calibration curve developed using the ΔR_ct_ (%) value of the EIS measurement result for the verification of the DNA aptamer that detects NS1 serotypes (S4, S1). ΔR_ct_ (%) was calculated as (R_ct_ (target) − R_ct_ (blank))/R_ct_ (blank) × 100%. It was confirmed that both blood types were detected, and the LOD (ng/mL) of serotypes 1 and 4 were measured as low concentrations of 0.025 and 0.022, respectively. Figure 3F displays the ΔR_ct_ value, indicating the detection selectivity of the undiluted human serum NS1-S4, NS1-S1, and DENV envelope proteins. Only the envelope protein exhibited a negative change in R_ct_, indicating excellent selectivity for DENV proteins. These results indicate that it may be a useful device for various clinical applications. The fabricated aptasensor for DENV detection suggested its potential as a promising tool for miniaturization and point-of-care devices.

Mills et al. developed a platform for the detection of two different DNA sequences using a single electrochemical sensor [90]. The sensor consisted of a universal stem-loop probe (USL) attached to a gold disk electrode and two analyte-specific adapter strands (m-ZIKV, f-ZIKV, m-DENV, and f-DENV), which were hybridized to a nucleic acid analyte in a four-way junction (4 WJ) structure to achieve specific binding with a high binding affinity. Figure 3H,I displays the calibration curves of the sensor for the response of current density j (μA/cm^2^) to the target ZIKV (T-ZIKV) and target DENV (T-DENV) concentrations (1 nM–75 nM). The area of the working electrode was measured using the cyclic amperometric method. The LOD was calculated as three times more than the value obtained by dividing the standard deviation of the blank electrode by the calibration curve slope. In Figure 3H, the current density according to the concentration of the T-ZIKV sequence was analyzed using m-ZIKV and f-ZIKV. The reaction time was 10 min, and it was confirmed that it increased linearly, with an LOD of 0.98 nM. As shown in Figure 3, the current density according to the concentration of the T-DENV sequence was analyzed using m-DENV-11 and f-DENV-19, and it was also confirmed that it increased linearly with a reaction time of 30 min. The LOD was 1.04 nM, which was slightly higher than that of ZIKV. Therefore, this study suggests that it is a promising general-purpose electrochemical sensor that can be used in the future using DNA sequences.

Therefore, electrochemical-based ZIKV and DENV biosensors using DNA nanotechnology present the possibility of clinical analysis with high selectivity and high sensitivity. However, further research is required for molecular analysis using real virus samples.

## 3. Electrical-Based Detection

Electrical sensors for virus detection have many advantages for in-field applications, such as high sensitivity down to the picomolar level, rapid detection without labeling processes, and portable readout circuits [91,92,93,94]. Electrical devices functionalized with receptors convert the electrical properties of target biomarkers into electrical signals such as electrical current, resistance, and capacitance. In some cases, electrical signals are intrinsically sensitive, with an exponential function of the concentration of the target biomarkers. Signal conversion is a label-free process that depends only on the intrinsic properties of the target biomarkers, which reduces the time required for sample preparation. The generated electrical signals can be measured and processed using small integrated circuits, enabling a handheld biosensor system.

In a field-effect transistor (FET), as a basic building unit of integrated circuits, an electrical potential applied to a gate controls the electrical current flowing from the drain to the source through a semiconducting channel. A similar operation occurs in FET-based biosensors. The electronic charges of virus biomarkers bound to the gate or channel surfaces modulate the electrical current or turn-on voltage, called the threshold voltage [95]. The change in the current or threshold voltage is then correlated with the amount of target analytes in the test solution.

**Figure 4:**
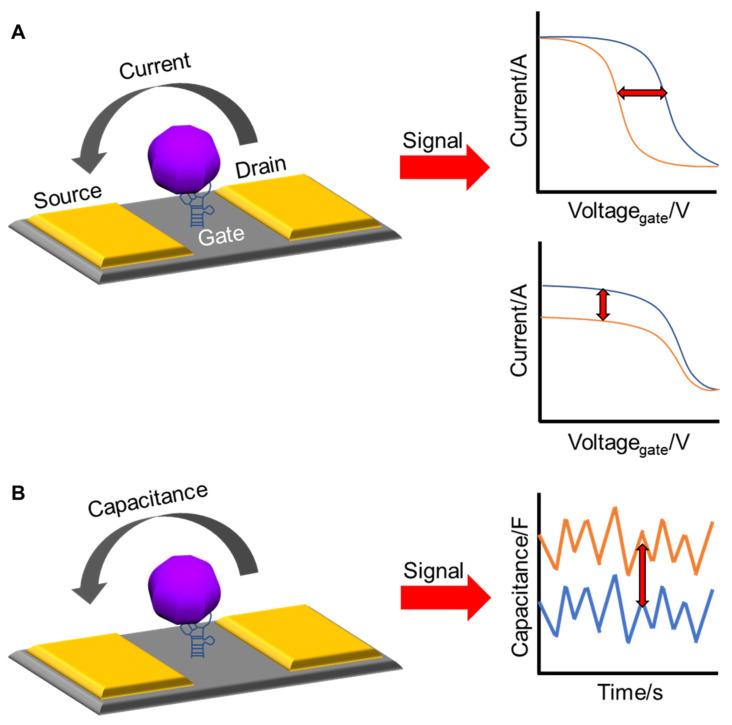
Schematics of (**A**) FET-based biosensor showing the change in voltage and current before and after bonding and (**B**) capacitive biosensor showing the change in capacitance before and after bonding.

A typical capacitor has two metal electrodes that are separated from each other by a certain distance to store electrical charges, producing a potential difference between the two electrodes. The capacitance (C) of the capacitor is presented as a measure with the Farad (F) unit, and is expressed as C = εε_0_A/d, where ε is the dielectric constant of the medium between the electrodes, ε_0_ is the permittivity of free space, A is the area of the electrodes, and d is the distance between the electrodes. In a capacitive biosensor, the specific binding of biomolecules inside the gap between the two metal electrodes results in a change in the capacitance by changing the dielectric constant or surface area of the electrodes. The capacitance of the biosensor is correlated with the number of viral biomarkers. Figure 4 shows a schematic of the electrical measurements.

Bioreceptors using DNA nanotechnology are useful for improving the detection capability of electrical biosensors, and are widely used as receptors for electrical biosensors to capture target DNA biomarkers. The self-assembled monolayer (SAM) process facilitates the immobilization of DNA receptors on the surface of field-effect transistors or capacitive biosensors. A target DNA biomarker with a strong negative charge on the backbone modulates the channel current of the FET-based biosensor in order to generate a strong signal. The conformational change of the aptamer upon binding to the target material can overcome the Debye screening effect that occurs when screening the charge of the target biomarker in a highly ionic solution [96]. This conformational change can bring the target biomarker closer to the channel surface, thereby increasing the effective charge of the channel to high current changes.

**Figure 5:**
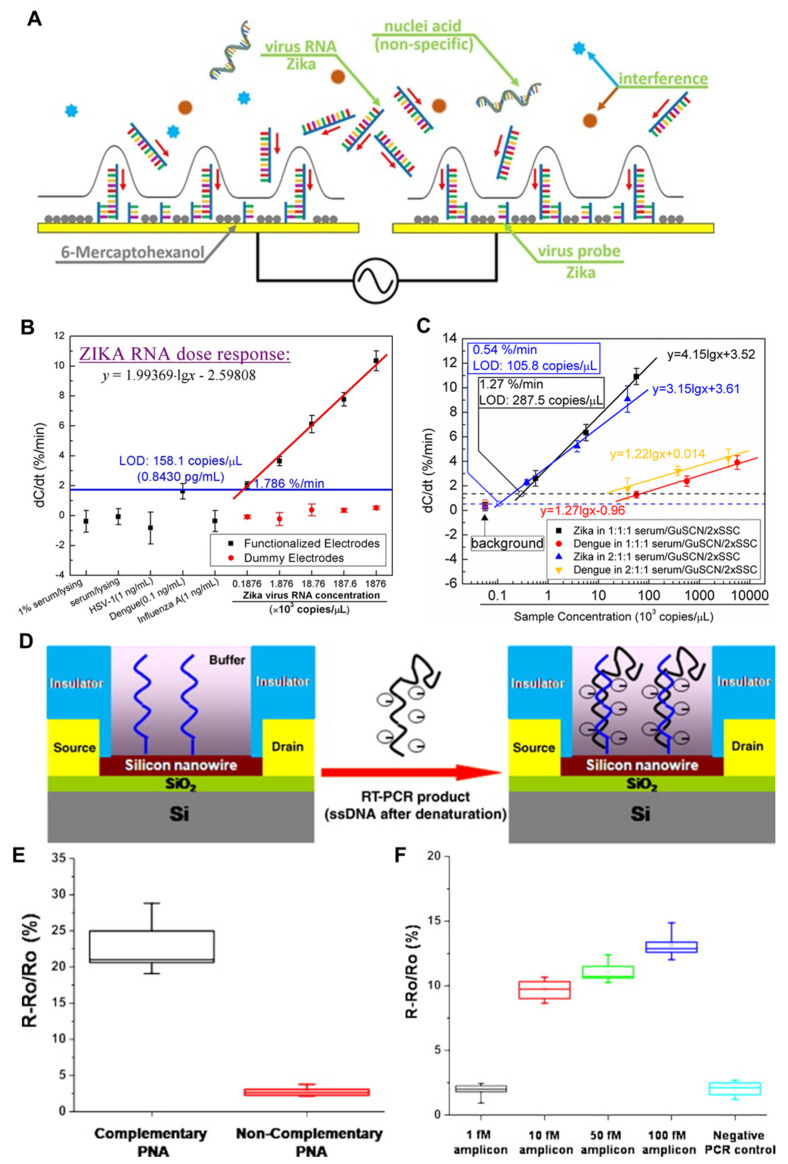
DNA−technology−based ZIKV and the DENV detection method using electrical method. (**A**) Conceptual drawing of ZIKV RNA affinity sensor with AC electrokinetics (ACEK) capacitive sensing. Analytes are attracted towards the electrode surface through ACEK effects. Specific binding between the functionalized ZIKV probe and ZIKV RNA causes a change at the interface (Cint), and the binding leads to a change in Cint, which is detected electrically using the same ACEK signal. Other particles, including influenza A virus, human herpesvirus virus 1 (HSV−1), DNA and DENV RNA, are considered non-specific interferences. (**B**) Responses of non-specific nucleic acid (HSV−1 and DENV) and virus (influenza A), and dose–response of ZIKV RNA spiked in serum/lysing solution. Reproduced with permission from [97], published by European Chemical Societies Publishing, 2017. (**C**) Dose responses of ZIKV gRNA human serum samples in 1:1:1 and 2:1:1 mixtures of serum, Guanidine thiocyanate (GuSCN), and 2 × saline-sodium citrate (SSC) as hybridization buffers. ZIKV (target) and DENV (interference) gRNA sample concentrations were converted into the equivalent concentration in pure serum. Adapted with permission from [98]. (**D**) A schematic diagram of the RT−PCR product of DEN−2 hybridized to the PNA−functionalized silicon nanowire (SiNW) sensor. Because PNA is neutral, the resistance change of the SiNW sensor before and after hybridization is attributed to the introduction of the negatively charged DNA (RT−PCR product). (**E**) Specificity of the SiNW sensor for the RT−PCR product of DEN−2. The purified RT−PCR product was applied to the complementary and the non-complementary PNA-functionalized SiNW sensors, respectively. (**F**) Resistance change versus concentrations of the RT-PCR product of DEN−2. Varying concentrations of the RT−PCT product from 100 fM to 1 fM were applied to the PNA-functionalized SiNW sensor. Negative RT−PCR product was used as a control. Reproduced with permission from [100], published by Elsevier, 2010.

Cheng et al. reported the detection of ZIKV RNA using a capacitive biosensor consisting of low-cost interdigitated microelectrodes and a sequence-specific receptor [97]. The biosensor operation was based on AC electrokinetics in which the AC signal applied to the microelectrodes induces a microfluidic flow that causes ZIKV RNA to move towards the microelectrodes and hybridize with the immobilized receptor in a test buffer solution (Figure 5A). This ZIKV RNA sensor had a wide dynamic range of 1.0 pg/mL (187 copies/μL) to 10 ng/mL (1.87 × 106 copies/μL) and a LOD of 0.843 pg/mL (158.1 copies/μL) in 1% serum (Figure 5B). The same research group then optimized the conditions for the functionalization buffer for receptor preparation and the hybridization buffer to yield high sensitivity and specificity for the detection of ZIKV RNA [98]. The sensor reached a LOD of 105.8 copies/μL with high specificity against ZIKV RNA (Figure 5C). It is worthwhile to note that these biosensors could detect the ZIKV RNA in 30 s without the need of PCR, which is ideal for field applications in a limited resource setting. A capacitive biosensor can be fabricated simply and at low cost compared to a FET-based biosensor. The detection sensitivity of a capacitive biosensor will be further improved through porous electrodes with a high surface area that increases the charge capacity [99], demonstrating the trade-off between the sensor performance (i.e., detection sensitivity) and sensor cost raised by additional processes for nanostructures.

Zhang et al. demonstrated a silicon nanowire (SiNW) biosensor for the detection of the reverse-transcription polymerase chain reaction (RT-PCR) product of Dengue serotype 2 (DEN-2) [100]. SiNWs with a width of 50 nm were prepared using top-down semiconductor processes, including deep ultraviolet lithography, dry etching, and ion implantation. A specific peptide nucleic acid (PNA) was functionalized onto the SiNW surface as a receptor in order to capture the target RT-PCR product (Figure 5D). The binding of the RT-PCR product to the PNA receptor resulted in a resistance change in the SiNWs via a field effect (Figure 5E). The results indicated that the SiNW biosensor could detect 10 fM of the RT-PCR product within 30 min (Figure 5F). Despite the low limit of detection, an additional process of RT-PCR in this work may be a disadvantage to increase the cost for biosensing, which can be further resolved using a technical breakthrough based on a pretreatment-free electrical detection [59].

Nuzaihan et al. further improved the LOD to 2.0 fM by reducing the size of the SiNWs to 20 nm [101]. The researchers clearly demonstrated that the relative change in the current response of a SiNW increased as the SiNW width decreased from 1 μm to 20 nm, confirming the higher sensitivity with a higher surface-to-volume ratio of the SiNW. The SiNW biosensor could be reused multiple times (>5) by the dehybridization of DNA pairs on the SiNW surface with hot deionized water at 90 °C for 5 min. However, SiNW nanostructures achieved by a combination of electron-beam lithography (EBL) and plasma dry etching have high surface defects on the sidewalls that degrade the reliability of device operation due to defect-induced charge trapping [102]. It was found that other semiconducting nanomaterials, such as carbon nanotubes [103] and graphene [104], can complement SiNWs in FET-based biosensors for the detection of ZIKV/DENV. Nanomaterials with intrinsically high surface-to-volume ratios can overcome difficulties in SiNW production by providing simple fabrication processes without expensive semiconductor equipment, such as deep ultraviolet lithography and electron beam lithography, which are required to obtain small-sized SiNWs. In addition to one-gate FET-based biosensors, double-gate FET-based biosensors have been developed to improve detection sensitivity by adjusting the gate controllability to target biomolecules [105,106]. Specifically, it is not necessary for aggressive scaling down of silicon nanowires in the case of double-gate FET-based biosensors. Additionally, SiNWs can be fabricated through mature CMOS technology.

## 4. Optical-Based Detection

**Figure 6:**
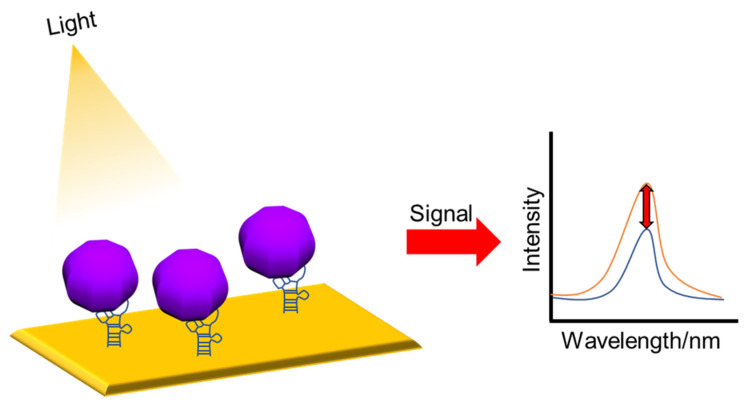
Schematic diagram of an optical sensor. Changes in intensity signals before and after binding.

Optical-based biosensors measure the absorption, reflection, and emission of optical properties by converting them to specific signals. This has advantages such as, it is cost-effective, has a small size, has high sensitivity and specificity, and can detect biological and chemical substances in real time without labeling. Optical-based biosensors utilize the interaction of an analyte with an optical field for optical detection and emit an optical signal that is directly proportional to the analyte concentration [57,107]. Figure 6 shows a schematic of the optical measurements. Quantum dots (QDs) can be used to detect fluorescence intensity. QDs include excellent light stability and optical performance, and have high quantum yields and long fluorescence lifetimes [108]. Recently, studies on the functionalization of QDs, using biomolecules such as antibodies, nucleic acids, and aptamers, have been conducted, and it is discussed that various biomarkers can be detected with high sensitivity [108]. Measurement methods can be divided into surface plasmon resonance (SPR)/localized surface plasmon resonance (LSPR) [61,109], Raman spectroscopy [110,111], and fluorescence [112,113]. In this section, we discuss DNA-nanotechnology-based biosensors for ZIKV and DENV detection using SPR/LSPR and fluorescence.

### 4.1. SPR/LSPR

The basic principle of SPR is to generate a surface plasmon wave, which is a phenomenon in which electrons vibrate on the surface when light is applied and collide with a metal. SPR and LSPR measure the adsorption of substances on the surface of metal nanoparticles. As the medium changes, the momentum of the plasmon changes, resulting in resonance, and the changes in the angle of incident light and refraction are measured accordingly [57]. In SPR, when a metal surface interacts with light rays, all the light propagates along the metal surface as an electric field without reflected light, whereas in LSPR, some of the incident photons are absorbed and some are scattered [114].

**Figure 7:**
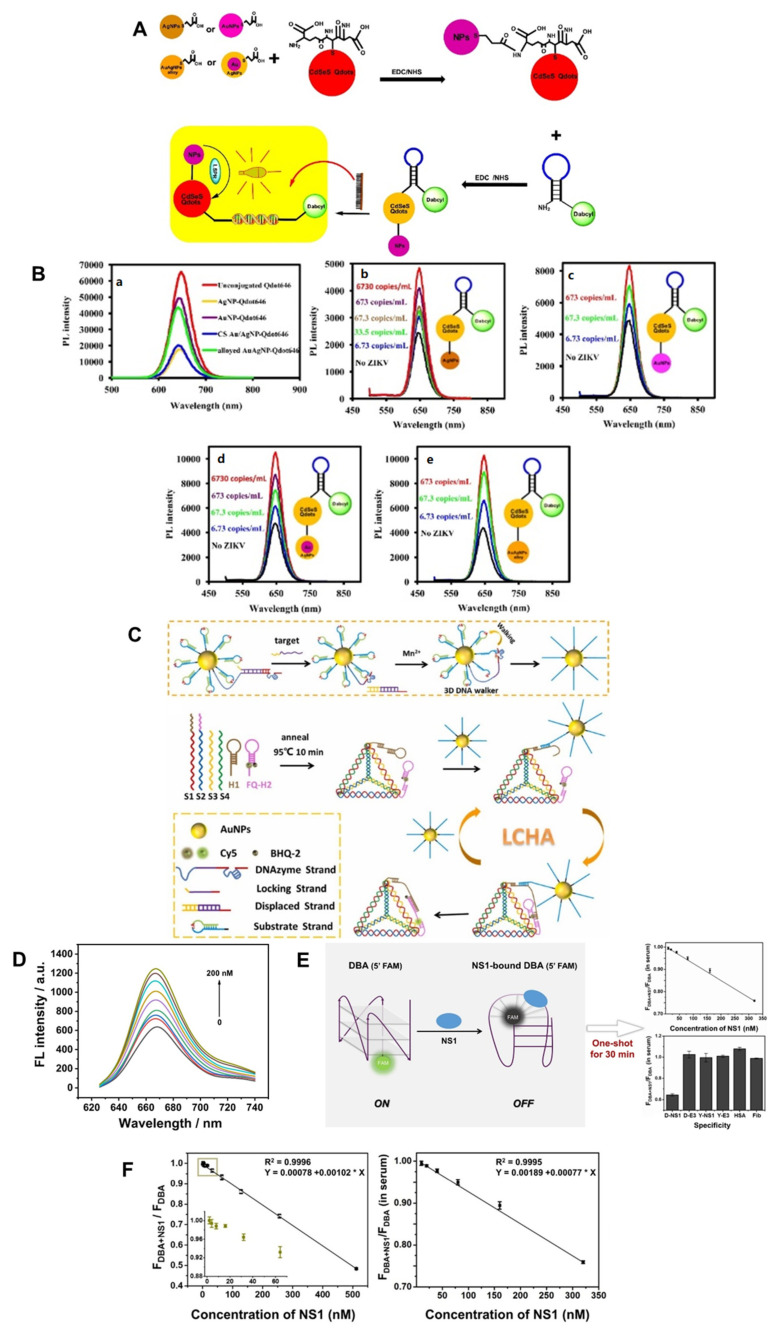
DNA-technology-based ZIKV and DENV detection method using optical method. (**A**) Schematic illustration of the conjugation of plasmonic NPs to GSH-CdSeS quantum dots (Qdot646). (**B**) (**a**) Effect of fluorescence quenching of plasmonic NPs on fluorescence of Qdot646 particles by type; (**b**–**e**) LSPR-mediated fluorescence enhancement of ZIKV RNA using type-specific plasmonic NP-Qdot-MB biosensor probes. Reproduced with permission from [115] published by Elsevier, 2017. (**C**) Schematic illustration of fluorescent biosensor for ZIKV detection using 3D DNA Walker part and LCHA part. (**D**) Changes in the FL spectrum of ZIKV according to the concentration of the fabricated biosensor. Reproduced with permission from [122], published by Elsevier, 2022. (**E**) Schematic illustration of the fabricated DENV detection fluorescence aptasensor. (**F**) Calibration curve of F_DBA+NS1_/F_DBA_ according to concentration change of DENV NS1 in buffer and 5% human serum. Reproduced with permission from [124], published by Elsevier, 2021.

Adegoke et al. developed a ZIKV RNA detection biosensor using a plasmonic nanoparticle (NP)-quantum dot (Qdot)-molecular beacon (MB) as a bioreceptor based on LSPR-mediated fluorescence signals [115]. The fluorescence intensity of the Qdots was mediated by the LSPR signal. The MB is an oligonucleotide hybridization probe that can detect the presence of a specific nucleic acid. It is a hairpin-shaped molecule with an internally quenched fluorophore that restores fluorescence upon binding to the target nucleic acid [116]. (AgNP), alloyed AuAgNPs, and bimetallic core/shell (CS) Au/AgNPs were synthesized and functionalized with 3-mercaptopropionic acid (MPA). Then, L-glutathione (GSH) was bound to the capped CdSeS alloy Qdots to form a fluorescent nanohybrid system. This was spliced into an MB loop designed to hybridize with ZIKV RNA and used as an ultrasensitive LSPR-fluorescence signal converter (Figure 7A). After hybridization of plasma NP-Qdot-MB and ZIKV RNA for 3 min, the LSPR-mediated fluorescence enhancement was confirmed according to the concentration of ZIKV (Figure 7B). LOD values for ZIKV RNA were alloyed AuAgNP-Qdot646-MB (LOD = 1.7 copies/mL) > CS Au/AgNP-Qdot646-MB (LOD = 2.4 copies/mL) > AuNP-Qdot646-MB (LOD = 2.9 copies/mL) > AgNP-Qdot646-MB (LOD = 7.6 copies/mL), and a bioreceptor that specifically detects ZIKV RNA and exhibits ultra-sensitive and excellent specificity was introduced.

In another study, Chowdhury et al. optimized a stable system by altering the distance-based LSPR between cadmium selenide tellurium sulfide fluorescent quantum dots (CdSeTeS QDs) and gold nanoparticles (AuNPs), resulting in the rapid and quantitative development of DENV serotypes (serotypes 1–4) [117]. Four nanoprobes were introduced using primer-probe serotype-specific hairpin single-stranded DNA (ssDNA) covalently linked to CdSeTeS QDs at different positions. The anchoring region of the hairpin complementary to each DENV serotype RNA was self-complementary by six polyguanines (poly-G) and polycytosine (poly-C), and one side was covalently bound to the CdSeTeS QDs. In addition, AuNPs functionalized with thiolated polyC were synthesized. Synthesized ssDNA or real RNA samples were used, and the target ssDNA/RNA sequence of each DENV serotype opened the complementary ssDNA loop sequence of the hairpin to form DNA/DNA or DNA/RNA hybridization. Accordingly, a linear strand of the ssDNA probe conjugated with the QD was formed, and the target DNA/RNA was aligned with the nanoprobe through complementary binding. LSPR generated from the surface resonance electrons of AuNPs has a strengthening or quenching effect on the fluorophore (quenching effect when it is near and a strengthening effect when it is far away) [118]. Fluorescence intensity was measured by combining the prepared nanoprobes with DENV ssDNA at various concentrations. LODs of 24.6 fM, 11.4 fM, 39.8 fM, and 39.7 fM were calculated for DENV ssDNA 1, 2, 3, and 4, respectively, and can be applied to actual DENV RNA. Using the distance-dependent LSPR phenomenon of fluorescent CdSeTeS QDs with adjacent AuNPs, a biosensor to detect DENV serotypes without amplification was reported for the first time.

### 4.2. Fluorescence

A fluorescence-based biosensor is based on the basic principle of visualizing the fluorescence signal that appears in a fluorescent dye, and the presence of an analyte is confirmed by a change in the intensity of the fluorescence signal [119]. Fluorescence-based biosensors have many advantages, such as good selectivity, high sensitivity, multiplex analysis, simple instrumentation, fast analysis time, and simple operation. Parameters, such as fluorescent tags, signal transducers, and analyte recognition devices must be carefully considered to obtain a better performance. In general, signal amplification techniques and brighter fluorescent tags are used to enhance fluorescence signals [120]. For example, Yang et al. established two nanomaterial-based surface-enhanced fluorescence strategies to link gold nanoparticles and silver nanoclusters to aptamers using brighter fluorescent tags [121]. Additionally, among signal amplification techniques, cyclic signal amplification is one of the most useful techniques. In this technique, the fluorescence signal is amplified many times to achieve a low detection limit and high sensitivity for biomolecules, increasing detection sensitivity and fluorescence signal [120].

A study conducted by Liang et al. proposed a fluorescent biosensor that integrates the localized catalytic hairpin assembly (LCHA) cascade amplification strategy and a DNA walker as a novel ZIKV assay platform for rapid, accurate, and low-cost diagnosis [122]. The catalytic hairpin assembly (CHA) reaction is an isothermal amplification strategy that is useful for amplifying and transducing signals to detect DNA and RNA, and the LCHA reaction provides a faster reaction rate [123]. As shown in Figure 7C, a DNAzyme-driven 3D DNA walker was constructed by assembling locked walking strands and a substrate hairpin probe to gold nanoparticles (AuNPs). The LCHA was designed by attaching hairpin DNA 1 (H1) and fluorophore-quencher-labeled hairpin DNA 2 (FQ-H2) to a DNA tetrahedron. The 3D DNA walker is triggered to release a working strand by the target ZIKV RNA sequence, which binds to the substrate hairpin and is cleaved in the presence of Mn^2+^. The working strand is then released from the DNA fragment and participates in subsequent binding and cleavage. By repeating the binding and cleavage processes, a large number of short-cleaved DNA segments linked to AuNPs can be generated. Next, a DNA segment linked to AuNPs was obtained and applied as an initiator of the LCHA reaction to recover the fluorescence. This biosensor obtained a stable signal within 2 h, and the fluorescence (FL) intensity result was obtained according to the concentration of ZIKV RNA (Figure 7D). These results indicate that the signal can be detected in the range of 50 pM to 200 nM ZIKV RNA and has a low LOD of 20 pM. This is because it is amplified by the 3D DNA walker and the LCHA reaction. Thus, in this study, a cost-effective and sensitive ZIKV detection fluorescent biosensor was developed.

In another study, Mok et al. developed a G-quadruplex (GQ)-based fluorescent aptasensor for one-shot detection of DENV NS1 [124]. GQ is a non-canonical four-stranded helix composed of four guanine planar G-tetrads stabilized by Hoogsteen hydrogen bonds [125]; GQ aptamers can self-assemble and are very stable, and recognition of their targets can cause conformational changes [126]. The guanine of the fluorophore-labeled GQ aptamer is oxidized more easily than the other nucleobases to quench the fluorescent dye. In this study, a DENV-derived NS1-binding aptamer (DBA), which forms a GQ structure, was introduced as a bioreceptor. 6-carboxyfluorescein (FAM) (5-‘FAM-DBA) was labeled at the 5′-terminus of DBA to fabricate a GQ-based fluorescent aptasensor in which structural changes occur via DENV NS1 (Figure 7E). The prepared aptasensor was incubated with NS1 at various concentrations for 30 min in order to observe the fluorescence intensity, and NS1 detection was quantified using the fluorescence ratio NS1-coupled DBA = F_DBA+NS1/_F_DBA_ (Figure 7F), where F_DBA+NS1_ is the fluorescence intensity of 5′ FAM-DBA bound to NS1 and F_DBA_ is the fluorescence intensity of DBA in NS1 storage buffer (PBS, pH 7.4). This aptasensor could detect DENV NS1 concentrations of 4 nM to 512 nM, with a LOD of 2.51 nM. Furthermore, DENV NS1 can be detected at a concentration of 2.81 nM to 360 nM in 5% human serum, with a LOD of 8.13 nM. Thus, this study reported the first DBA that forms a GQ structure and exhibits conformational changes mediated by DENV NS1.

These studies have excellent sensitivity and specificity, and were the first to report the technology. However, fluorescent substances are used for bioreceptors, and most of them contain many substances. In such cases, the structure or function of the biomolecule may be altered and the experimental results may be distorted. Therefore, it is necessary to focus on the development of biosensors with simple constituent materials.

## 5. Conclusions

In this review, the authors examined the progress in the manufacturing of ZIKV and DENV detection biosensors using DNA nanotechnology (Table 1). In addition to aptamers, bioreceptors use various DNA nanotechnologies, such as primers, PNA, molecular beacons, and DNAzymes. In addition, these techniques can be applied to future research. Currently, diagnostic devices using antibodies are commercialized, which is a great economic burden. Using DNA nanotechnology, it is easy to introduce a new bioreceptor as the target, such as the protein or nucleic acid of a virus. This technology is also used to detect other targets, such as toxins or biomarkers other than viruses, and the performance is also excellent [59,127]. However, compared to antibodies, the selectivity for a target and the accuracy of diagnosis are lacking. In addition, most of the diagnostic devices using antibodies in daily life are up to date. Various studies are needed to popularize this, so the development of biosensors that detect ZIKV and DENV based on various DNA nanotechnologies will increase in the future. Recently, research on electrochemistry and electricity-based biosensors using DNA nanotechnology has been increasing; however, studies associated with optical-based biosensors are declining. As can be seen from this review, the bioreceptors of optical-based biosensors contain many materials, while the bioreceptors of electrochemical and electricity-based biosensors are relatively simple. The introduction of a simple bioreceptor is economically important. Biosensors based on DNA nanotechnology have many advantages over existing detection technologies; therefore, they are promising and have strong potential. These biosensors can be used as a tool to control the spread by diagnosing ZIKV and DENV in South America. In addition, when a sudden viral pandemic happens, such as the COVID-19 pandemic, it will be possible to respond quickly by rapidly producing DNA-nanotechnology-based diagnostic devices. In this review, we have demonstrated that DNA-nanotechnology-based biosensors can be used to detect ZIKV and DENV in samples from infected patients. In addition, since ZIKV and DENV have similar symptoms, there may be confusion, and cross-infection between ZIKV and DENV should be confirmed. Therefore, additional research on biosensors for the simultaneous detection of ZIKV and DENV is required.

## Figures and Tables

**Table 1:** ZIKV and DENV detection biosensors based on DNA nanotechnology.

Detection Method	Target	DNA Nanotechnology	Detection Range	LOD	Detection Time	Ref.
Electrochemical	ZIKV	primer	25 nM–340 nM	25 nM	90 min	[88]
		ssDNA	84.0 pM–1.41 nM	25.4 pM	6 h	[128]
		cDNA	0.3 nM–3 μM	0.1 nM	125 min	[129]
		ssDNA	1.322 pM–13.22 nM	1.322 pM	20 min	[130]
	DENV	Aptamer	1.667 pM–16.67 nM, 1.667 pM–166.7 nM	4.168 pM, 3.667 pM	30 min	[89]
		ssDNA, cDNA	100 fM–1 nM	100 fM	10 min	[131]
		ssDNA	100 pM–100 μM	100 pM	30 min	[132]
		crRNA, hairpin DNA	5 fM–50 nM	0.78 fM	80 min	[133]
		ssDNA	1 nM–100 nM	430 nM	2 h	[134]
	ZIKV, DENV	USL	1 nM–75 nM	0.98 nM, 1.04 nM	10 min	[90]
Electrical	ZIKV	ssDNA	13.22 fM–132.2 pM	11.15 fM	30 s	[97]
		ssDNA	13.22 fM–1.322 pM	7.48 fM	30 s	[98]
		Aptamer	100 pM–10 μM	38.14 pM	10 s	[135]
		Aptamer	0.208 fM–20.8 pM	0.208 fM	-	[45]
	DENV	PNA	10 fM –100 fM	10 fM	30 min	[100]
		ssDNA	10 fM–10 μM	2.0 fM	Overnight	[101]
		ssDNA	100 fM–1 nM	198.5 fM	2 h	[136]
Optical	ZIKV	MB	0.0038 fM–3.843 fM	0.0013 fM	3 min	[115]
		hairpin DNA	50 pM–200 nM	20 pM	2 h	[122]
		primer	0.1 nM–10 nM, 0.5 nM–7 nM	32 pM, 9 pM	30 min	[27]
	ZIKV, DENV	PNA	3.3 nM–40 nM	3.3 nM	1 h	[137]
	DENV	hairpin ssDNA, primer	1 fM–100 pM	11.4 fM	2 min	[117]
		Aptamer	10 nM–320 nM	8.13 nM	30 min	[124]
		ssDNA	0.125 nM–6.25 nM	0.125 nM	20 min	[138]
		ssDNA	1 fM–1 mM	1.21 fM	15 min	[139]

## Data Availability

Data sharing not applicable to this article.
